# Enrollment in community based health insurance program and the associated factors among households in Boricha district, Sidama Zone, Southern Ethiopia; a cross-sectional study

**DOI:** 10.1371/journal.pone.0234028

**Published:** 2020-06-02

**Authors:** Dawit Nageso, Kebede Tefera, Keneni Gutema

**Affiliations:** 1 Boricha District Health Office, Balela, Sidama, Ethiopia; 2 School of Public Health, College of Medicine and Health Sciences, Hawassa University, Hawassa, Ethiopia; Ministry of Health and Sports, MYANMAR

## Abstract

**Background:**

In absence of any form of health insurance, out-of-pocket payments for health care lead to decreased use of health services and catastrophic health expenditures. Community-based health insurances has been promised financial model for informal sectors to reduce these problems in many countries. When this comes down to Ethiopia, in the South Nation Nationality People’s Region of the country established 52 schemes including Boricha district, the study area However, there has been little evidence about the enrollment status and the associated factors in the study area in particular elsewhere in general.

**Objective:**

The study aims to assess the current enrollment status of households in community based health insurance and the associated factors in Boricha district of Sidama Zone, Southern Ethiopia.

**Methods and materials:**

A community based cross-sectional study design was employed from February 01, 2019 to March 31, 2019, using a sample of 632 households. Data were collected using interviewer-administered pre-tested questionnaire and entered into EPI-Info 7and transported to SPSSversion20 for analysis. Multi-variable logistic regression analysis along with odds ratio and the corresponding 95% CI was conducted and significance was declared at P-value <0.05.

**Results:**

Current enrollment status of households in community based health insurance was found to be 81 (12.8%). According to this study, educational status; secondary school& above[AOR = 2.749, 95%CI(1.142, 6.618)], timing of collecting premium [AOR = 0.433; 95% CI (0.196, 0.958)], family size ≥5, [AOR = 4.16;95%CI (1.337, 12.944)], no trust on scheme management[AOR = 0.272; 95%CI (0.140, 0.528)], lack of information [AOR = 0.086; 95%CI (0.026, 0.288)], dissatisfaction with health care service received[AOR = 0.303; 95%CI (0.171, 0.537)], no chronic illness in the family[AOR = 0.259; 95%C.I.(0.137, 0.488)] were factors significantly associated with current enrollment status in CBHI.

**Conclusions:**

Households head’s education status, timing of premium collection, family size, no trust on scheme management, lack of information, services dissatisfaction and chronic illness in the family member were the identified factors associated with enrollment in CBHI in the study area. Therefore, to enhance the enrollment and sustainability of CBHI in the study area awareness creation, improving timing of premium collection, strengthening scheme management, improving quality of service are the areas that decision makers needs to intervene.

## Introduction

In countries where limited health insurance are practiced, direct out-of-pocket (OOP) payments dominate health care financing system, this is mainly the case of many low-and middle income countries [1]. Thus, many households in these countries lack adequate financial protection; households face financial catastrophe and its impoverishing effects of paying for health services in the form of out-of-pocket [2]. Globally, about 44 million households faced catastrophic health expenditure while about 25 million households are impoverished because of direct health care payments. Over 90% of healthcare financial difficulties and their consequences have been occurring in Sub-Saharan African countries, where resources are limited [2]. Thus, implementation of community-based health insurance (CBHI) has emerged as a possible health financing mechanism in reducing out of pocket payment, particularly in areas where many people engage in informal workers and rural residence of these countries [3]. CBHI is known for facilitating resource mobilization to promote health service uptake and provide financial risk protection to insured members by reducing their OOP payments [4]. In the absence of any form of risk-pooling mechanism, high OOP payments can lead to a high level of poverty and catastrophic financial stress in vulnerable households. In such situations they are forced to borrow money, sell assets, or change resources from other necessity to cover healthcare expenditures [5–7]. Historically, CBHI has been implemented in many countries of the worlds’ health financing system, but it is particularly prevalent in Sub-Saharan Africa (SSA). Especially, it is part of an overall health financing strategy in a number of countries where the high OOP financing of care, the doubtful financial flows from donors, the large rural and informal sector populations, and the weak capacity of governments to raise taxes [8]. In Ethiopia, CBHIS first launched in June 2011 as pilot in 13 districts found in four main regions namely, Tigray, Amahara, Oromiya and South Nations and Nationalities people’s Region (SNNPR) [9]. It was set up as a community-based health project that gathers payments made by members into a fund that covers basic health care costs at local health care centers whenever any member of the group is sick [10]. High proportion of enrolled households in CBHI is an indicator of the general attractiveness of scheme and also it measures the extent to which the scheme sustained. The higher the enrollment in health insurance, the more households can avoid the financial implications of treatments costs and secure access to care when it is needed. In practice, however, CBHI often fails to achieve its potential, primarily because it fails to high levels of participation [11].

Previous study focused on the already enrolled households’ level of satisfaction with CBHI scheme and the associated factors and such study provide significant contribution for the implementation and further expansion of the program [12]. However, less is known of the enrollment status and factors associated with it. Consequently, the available report shows low coverage in which the current study wants to address. For instance, one national report in Ethiopia shows only 48% enrollment of households in the pilot schemes with large variation within and between districts. It ranges from as low as 25% in Deder and as high as 100%(Universal enrollment) in Yirgalem town [13].

Regardless of the promising government’s health financing strategy in that the scheme can be expanded and that CBHI can provide an important basis for reducing out-of-pocket payments, researches on the households enrollment in CBHI program and the associated factors are still very limited in Ethiopia in general and in the Boricha district of south Ethiopia in particular. Thus, the current study aims to examine enrollment status and the associated factors in the study area to fill the aforementioned gaps.

## Methods and materials

### Study area and period

The study was conducted in Boricha district, Sidama Zone, Southern Ethiopia from February to March, 2019. Boricha district is one of the 19 rural districts and 6 town administrations in the Zone; it is located 32 km from Hawassa town, the capital of SNNPR. It was among the five districts of Sidama Zone where the CBHI scheme was introduced as the expansion of pilot program next to Yirgalem town in Sidama zone. The total estimated population of the district was 332,791 as of mid-2018. The district has a total of 42 Kebeles, of which municipal town Kebeles were 03 and rural Kebeles were 39. The total estimated households of the district were 67,916. CBHI was implemented in all Kebeles of the district in 2012/13. Regarding health care facilities, there were one public district Hospital, 10 public health centers, one None Governmental Organization (NGO) clinic, 39 health posts and eight private clinics in the district [14].

### Study design

A community based cross-sectional study design was employed.

### Source and study populations

The source populations for the study were all households reside in Boricha district. Households’ heads older than 18 years who are not involved in the formal sector employment and reside for 6 months in the selected Kebeles of the district were included in the study.

### Sample size determination

The sample size was calculated using single population proportion formula by considering the following assumptions: n = Z^2^_α/2PQ_/d^2^, Z_α/2_ = Reliability Coefficient with 95% confidence interval, d = standard error allowed (0.05) and p = proportion of household enrolment in CBHI = 43.2% from previous study [15]. Considering design effect (DE) of 1.5, and non-response rate of 10%, the calculated total sample size was 622 households assuming this sample for determining the level of enrollment status. However, it is necessary to calculate sample size for factors associated with enrollment status of households. Hence, we calculated based on previous studies; such as members low confidence on CBHI management, information about CBHI and family size using EpiInfo statCalc; with the assumptions percentage outcome in unexposed group, power of 80%, Ratio (Unexposed: Exposed) one (1), 95%CI, Odds ratio, non-respondent rate (10%) and design effect (1.5). Finally, we have taken lack of trust on CBHI management as a main associate factors with enrollment status of households in CBHI 26.1% of outcome in unexposed group, Odds ratio of 1.88 which yielded the largest sample size, 392 [15]. Considering 10% non-response rate, the final total sample in this case was size was 646. Hence 646 was larger, we considered it as our final sample size for the study.

### Sampling procedures

First the Kebles were stratified in to Urban and rural. Then, a two stage sampling procedure was employed. Using simple random sampling technique, one (01) Kebele from urban and ten Kebeles from rural residence were taken. Then, the sampled 646 households were selected using stratified systematic random sampling technique after assigning proportionate amount of households to each sampled Kebeles.

### Data collection tools and procedure

Data were collected using interviewer-administered pre-tested questionnaire. The questionnaire was adapted from national Health Insurance Agency’s CBHI evaluation survey of Ethiopia [16] and adjusted in to local context. The English version of the adapted questionnaire was further translated in to local language (Sidama) for data collection. The tool was pre-tested on 5% of the actual sample size in two Kebeles different from target area but have similar socio-demographic status and other related variables with the study population. Based on the pre-test findings, some items were revised, edited and some ambiguous items were clarified. Consequently, information drawn from the pretest was discussed among data collectors and supervisors in order to ensure better understanding of data collection process. Five diploma nurses who are native speakers of Sidama language and two professional supervisors with BSc. in health were participated. Data were collected by face to face interview after introducing the study objectives to the study participants and coming to consensus on the importance of the study.

### Data quality assurance techniques

To assure the data quality, training was given for data collectors and supervisors for 02 days. Each item of data collection tool was seen thoroughly and clarified during the training. Besides, pre-testing of the questionnaire was done to improve the validity and reliability of the instrument. Further, appropriate information and instruction on the objective and relevance of the study were given for the respondents. Proper categorization and coding of responses and skip patterns were also maintained. Questionnaires filled by data collectors were reviewed and checked daily for completeness, consistency and relevance by the supervisors and then by principal investigator. All the necessary feedback was offered to data collectors in the next morning before the routine data collection was conducted.

### Data processing and analysis

Data entry and processing were done by EPI INFO version 7 computer packages and exported to Statistical Package for Social Sciences (SPSS) version 20 for analysis. Descriptive statistics including frequency, percent, table and graph were used to describe the results. The Hosmer- Lemeshow statistics and deviance coefficient was used to check the goodness of fit of the model and the model was good fit (P = 0.863), and also multi-collinearity was checked for variables that were statically significant on bivariate analysis; accordingly variables with high correlation coefficient were not found in the model. Similarly, missing values were also checked. Factors associated with current enrollment status of households in CBHI were first assessed using binary logistic regression. Based on this assumption, we considered variables with P ≤0.25 cutoff point as a candidate for our multivariable analysis. Wealth status of the households was computed by Principal Component Analysis (PCA). Finally, a P-value of ≤0.05 was considered to declare the significant factors along with Odds ratio and the correspondent 95%CI.

### Ethical consideration

Ethical clearance for the study was obtained from Hawassa University institutional Review Board (IRB). Also, permission letters were taken from Hawassa university school of public health, Sidama Zone Health Department and Boricha District Health Office. To maintain confidentiality, individual identifiers were not included. Moreover, a well explained informed consent was obtained from each respondent. Participants were also given right to participate or not, answer all questions or stop any were before completing all questions.

### Operational definition

**Community based health insurance:** is an insurance scheme arranged for informal sector, managed and operated by governmental structure that provides risk pooling to cover all or part of the costs of health care services [15].

**Catastrophic health expenditures**: is an expenditure related to medical treatment that can pose as treat towards a households financial ability to maintain it subsistence needs (minimal resources that are necessary for survival [17].

**Enrollment status in CBHIS:** is acceptance of CBHI to use and, pay premium for a complete year and possess updated service card [15].

**Health insurance**: is insurance against the risk of incurring medical expenses among individuals and families [15].

**Kebele:** is smallest administrative structure in Ethiopia.

**Out-of-pocket payment: is** type of health service cost that covered by service users to the service providers at a time and place of service provision [8].

**Revenue collection**: is process by which health system obtains financial contributions from different bodies [8].

**Pooling:** is spreading the risk of health expenditures among all members through accumulated and managed contributions from individuals [8].

**Purchasing:** is process by which pooled contributions are used to pay providers to deliver a set of health interventions [8].

**Household satisfaction: -**the satisfaction of households with the service provided in the health facility by health professionals were measured with 8 questions. They were organized to be responded in the Likert scale, ranging from 1(Poor) to 5(Excellent). The, the mean value was calculated and those who score above the mean were considered to be satisfied and those who score below the mean were considered to be dissatisfied [18].

**Time taken to reach nearby facility**: it is time taken when an individual uses on foot to go to a nearby health facility.

## Results

Out of the 646 respondents who were identified for the study, 632 were responded to the interview, yielding 97.8% response rate.

According to this finding, only 81 (12.8%) of households were currently enrolled in the community based health insurance. The study also revealed factors such as educational status of households’ head, inconvenience of time of premium collection, family size, trust on scheme management, having information about CBHI, satisfaction with the service provided by nearby facilities and chronic illness in the family member were associated with current enrollment status in CBHI.

### Socio-economic and demographic characteristics of the respondents

From the total study participants, 375 (59.3%) were males and 257(40.7%) were females. Considering the age of participants, 336 (53.2%) were between 18 and 38 years, 260 (41.1%) were between 39 and 58 and the rest 36 (5.7%) were 59 years and above. Three hundred sixteen (50%) of the respondents were illiterate, 141 (22.3%) of the study participants didn’t attend formal education, but because of adult education they can read and write, and the primary, and secondary and above account for 120 (19%) and 55 (8.7%) respectively (Table 1).

**Table 1:** Demographic and socio-economic characteristics of respondents in Boricha district, Southern Ethiopia, 2019 (n = 632).

Variables	Category	Frequency	Percent
**Gender of household head**	Male	375	59.3
	Female	257	40.7
**Age of respondents**	Age 18–38	336	53.2
	Age 39–58	260	41.1
	Age above 59	36	5.7
**Marital status of respondent**	Married	529	83.7
	Single	85	13.4
	Others	18	2.8
**Educational status**	Illiterates	316	50.0
	Can read & write	141	22.3
	Primary	120	19.0
	Secondary & above	55	8.7
**Family size of household**	<5 member	83	13.1
	> = 5 member	549	86.9
**Occupation of household head**	Farmer	569	90.0
	Merchant	63	10.0
**Residence of households**	Rural	602	95.3
	Urban	30	4.7
**Wealth quintile of households**	poor	252	39.9
	medium	127	20.1
	rich	253	40.0
**Time to reach nearby facility**	<30 min. near to reach	160	25.3
	30–60 min. medium	255	40.3
	>60 min. far to reach	216	34.2
**Money shortage**	No	463	73.3
	Yes	169	26.7

Regarding occupation of household head, the majority were farmers 569 (90%) and the rest 63 (10%) were merchants. In considering residence of respondents, about 602(95.3%) were from rural and only 30 (4.75) were Urban. Concerning marital status of study subjects, 529 (83.7%) were ever married, 85 (13.4%) single and 18 (2.8%) others. Five hundred forty nine (86.9%) of respondents had five or more family members and the rest 83 (13.1%) had less than five family members (Table 1).

With reference to accessibility to health facilities in terms of time it takes to reach it, about 160(25.3%) of the study participants traveled for <30minutes, 255(40.3%) respondents travel for 30–60 minutes and 216 (34.2%) travel more than 60 minutes to get health care services when required. Concerning Wealth index of individual household, 252 (39.9%), 127 (20.1%) and253 (40%) of the households were of poor, medium and rich respectively (Table 1).

### Current enrollment status of household in community based health insurance in Boricha district South Ethiopia, 2019 (n = 632)

**Fig 1:**
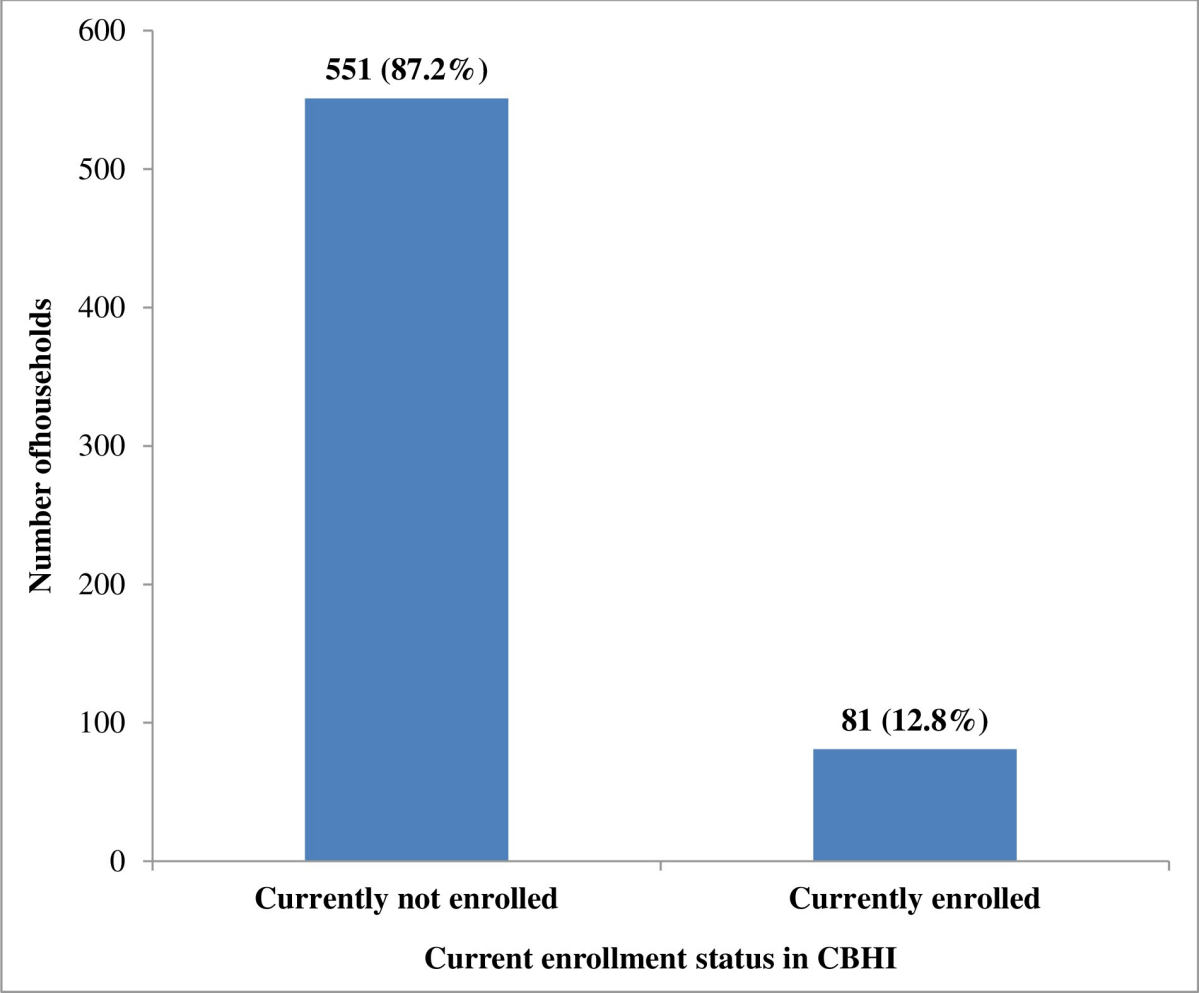
Enrollment status of households in CBHI in Boricha district, Southern Ethiopia, 2019.

Out of the total 632 study participants, 551 (87.2%) were currently not enrolled in community based health insurance (CBHI) and only 81 (12.8%) were enrolled in CBHI currently **(Fig 1).**

### Scheme related factors

In this study, out of the total respondents, 88 (13.9%) said that they waited long time to get the service card after paying, 485 (76.7%) ever heard about CBHI scheme,130 (20.6%) yearly based payment regardless of getting service,182 (28.8%) said they received poor service as compared to payers, 98 (15.5%) said payment is high compared to payers, 165(26.1%) complained inconvenience of premium collecting time, 98 (15.5%) accidental change of rule/regulations by the scheme management, 235 (37.2%) said they have no trust on scheme management and 94 (14.9%) reported inappropriate use of service card (Table 2).

**Table 2:** Participants’ response about features related with community based health insurance in Boricha district, 2019(n = 632).

Variables	Categories	Frequency	Percent
**Having information about CBHI**	No	147	23.3
	Yes	485	76.7
**Long time waiting service card after paying**	No	544	86.1
	Yes	88	13.9
**Year based payment without served**	No	502	79.4
	Yes	130	20.6
**poor service as compared to payers**	No	450	71.2
	Yes	182	28.8
**High payment as compared to OOP**	No	534	84.5
	Yes	98	15.5
**Inconvenience of premium collecting time**	No	467	73.9
	Yes	165	26.1
**Accidental change of rule**	No	534	84.5
	Yes	98	15.5
**no trust on scheme management**	No	397	62.8
	Yes	235	37.2
**In appropriate use of service card**	No	538	85.1
	Yes	94	14.9

Health related factors affecting current enrollment status in CBHI in Boricha district, 2019

All study participants interviewed on potential health related factors. Accordingly, five hundred seventeen (81.8%) said that they wait 60–360 minutes& above, 115 (18.2%) noted that they wait for ≤60 minutes. Seventy three (11.6%) of the respondents claimed that they received poor service in governmental health care facilities as compared to private ones. Ninety four (14.9%) of the respondents said they experienced chronic illness in their family members, 455 (72%) were not satisfied in the service they received from the nearby facility and 486 (76.9%) reported shortage of drugs in the health care facilities they visited (Table 3).

**Table 3:** Health related factors affecting current enrollment in community based health insurance in Boricha district, 2019.

Variables	Categories	Frequency	Percent
Waiting time	< = 60 minutes	115	18.2
	60–360 minutes & above	517	81.8
Poor serve in governmental institutions	No	559	88.4
	Yes	73	11.6
Chronic illness in the family	No	538	85.1
	Yes	94	14.9
Satisfaction of service in the nearby facility	Satisfied	177	28.0
	Dissatisfied	455	72.0
Drug availability	Insufficiently available	486	76.9
	Sufficiently available	146	23.1

### Determinants of enrollment status of household in community based health insurance program

In multivariable analysis, the significant factors associated with CBHI enrollment (P<0.05) were; educational status, family size, having information about CBHI scheme, timing of collecting premium, lack of trust on scheme management, dissatisfaction in the service provided by nearby health facilities and chronic illness experiences in the family (Table 4).

**Table 4:** Multivariable analysis result on factors associated with enrollment in community based health insurance in Boricha district, 2019 (n = 632).

Variables	Enrollment status		COR(95%CI)	AOR(95% CI)
	Yes	No		
**Educational status**				
Can read and write	13(16%)	128(23.2%)	0.842(0.430,1.650)	0.983(0.452,2.139)
Primary	22(27.2%)	98(17.8%)	1.862(1.0339,3.337)	1.882(0.920,3.851)
Secondary and above	12(14.8%)	43(7.8%)	2.315(1.113,4.813)*	2.749(1.142,6.618)*
Illiterates	34(42%)	282(51.2%)	1	1
**Family size of household**				
≤5 members	77(95.1%)	472(85.7%)	3.222(1.147,9.052)*	4.16(1.337,12.944)*
>5 members	4(4.9%)	79(14.3%)	1	1
**Time to reach nearby health facility**				
30–60 minutes	18(22.2%)	237(43.1%)	0.343(0.184,0.641)*	0.480(0.229,1.005)
≥60 minutes	34(42%)	182(33.2%)	0.844(0.490,1.434)	0.995(0.521,1.899)
<30 minutes	29(35%)	131(23.8)	1	1
**Having information about CBHI**				
No	3(3.7%)	144(26.1%)	0.109(0.034,0.350)*	0.086(0.026,0.288)*
Yes	78(96.3%)	407(73.9%)	1	1
**Shortage of money**				
Yes	12(14.8%)	157(28.5%)	0.436(0.230,0.828)*	0.769(0.344,1.721)
No	69(85.2%)	394(71.5%)	1	1
**Inconvenience of premium collecting time**				
Yes	9(11.1%)	156(28.3%)	0.317(0.154,0.648)*	0.433(0.196,0.958)*
No	72(88.9%)	395(71.7%)	1	1
**No trust on scheme management**				
Yes	14(17.3%)	221(40.1%)	0.312(0.171,0.569)**	0.272(0.140,0.528)**
No	67(82.7%)	330(59.9%)	1	1
**Waiting service card for long time**				
Yes	3(3.7%)	85(15.4%)	0.211(0.065,0.684)*	0.350>(0.100,1.21)
No	78(96.3)	466(84.6%)	1	1
**Drug availability in facility**				
Insufficient available	53 (65.4%)	433 (78.6%)	0.516 (0.313.0.851)*	0.717 (0.376, 1.366)
sufficient available	35 (43.2%)	118 (21.4%)	1	1
**Satisfaction on services**				
Dissatisfied	43(53.1%)	412(74.8%)	0.382(0.237,0.615)**	0.303(0.171,0.537)**
Satisfied	38(46.9%)	139(25.2%)	1	1
**Chronic illness in the family**				
No	14 (17.3%)	221 (40.1%)	0.298 (0.175,0.507)**	0.259 (0.137, 0.488)**
Yes	67 (82.7%)	330 (59.9%)	1	1

Accordingly, participants with educational status of secondary& above were almost 3 times more likely to be enrolled than those who were illiterates, AOR = 2.749; 95%CI (1.142,6.618). Enrollment status of households in CBHI among participants who had family size ≥5 was about 4 times higher than those who had family size <5, AOR = 4.16;95%CI (1.337, 12.944).

Consequently, Household heads who did not trust scheme management were enrolled less likely than who did and this was significant, AOR = 0.272; 95%CI (0.140, 0.528). Similarly, CBHI enrollment among respondents who were not informed about it were less likely than those who were informed, AOR = 0.086; 95%CI (0.026, 0.288) (Table 4).

Subsequently, enrollment status in CBHI among respondents who complained timing of collecting premium were significantly less likely than those who did not, AOR = 0.433; 95%CI (0.196, 0.958**).** Household’s enrollment in CBHI among respondents who were dissatisfied by services in nearby facility were also significantly less likely than those who were satisfied, AOR = 0.303; 95%CI (0.171, 0.537).

Similarly, enrollment status of households in CBHI among study participants who didn’t experienced chronic illness in their family member were significantly less likely than families who experienced chronic illness in their family, AOR = 0.259; 95%C.I. (0.137, 0.488) **(**Table 4).

## Discussion

As compared to previous studies (18–20), CBHI enrollment status in the current study was very low. For instance, the study done in Southwest Ethiopia revealed 77.85% enrollment rate [19]. Similarly, study conducted in Aleltu district of Oromia region in Ethiopia and another in Nigeria showed 75% [20] and 62.8% [21] respectively.

The possible reason for this discrepancy might be that the enrollment status measured in the current study considered acceptance of CBHI to use, and pay premium for a complete year and possess updated service card. For example, members who ever enrolled but drop out were not included in this study. Besides, variations in commitment of local decision makers might also have resulted in the low coverage of CBHI scheme reported in our study area.

According to the findings of the current study, educational status of the households’ head was among a statistically significant factors associated with the current enrollment in CBHI. Households whose heads were secondary & above were almost three times higher to be enrolled in CBHI than the households’ heads that were illiterate. This is in line with previous studies [21, 22]. As is expected, education influence people’s knowledge, attitude and practice for certain rewarding programs like that of being CBHI voluntary membership.

In support of previous studies [23, 24], in our study, household heads who had never experienced chronic illness in their family were less likely to be enrolled in CBHI than households whose family members experienced chronic illness. This positive association between the two might have occurred due to the fact that high-risk individuals usually prefers to be insured than the low risk or healthy individuals to avoid financial risk of out of pocket payment for health care services. This might be an indication of the likely risk of adverse selection.

In this study households with larger family sizes were more likely to be enrolled in CBHI than household with smaller family size. This finding is similar with the finding of national evaluative study conducted in Ethiopia [25], and also in line with the study done in rural community of Fogera District, North West Ethiopia [26]. Obviously, households with large family size faces higher risk of being sick and expected to suffer from financial risks, particularly in low income communities. So, they might have chooses to be enrolled to avoid the risk of out of pocket payment during the time of illness.

In our study participants who have do not trust scheme management were less likely to be enrolled in CBHI than those who trusted scheme management similar to previous studies [21, 27].The scheme administrators might have not been responsive to control and support the scheme in relation to community’s preference, people’s overall satisfaction and trust with the CBHI is likely to decrease, In turn, this affects enrollment in CBHI even more highly.

Participants who complained inconvenience of premium collecting time were less likely to be enrolled in CBHI than those who did not. This is in line with previous reports in one study on [20, 27], and also in one study on CBHI in developing countries [21]. Possible explanation for this might be; informal sectors usually rural communities are characterized by low saving practice that make them only capable to pay at specific point in time, for instance during harvesting time(seasonal based income). As a result, they may not have the cash in pocket to pay as scheduled by premium administrators.

The current study showed that being informed about scheme is significantly associated with enrollment in CBHI. This is in support of a study done in Nigeria [28]. As is expected, individuals with better information may ask details of the services and get more understandings of its advantage that drives them to be enrolled in CBHI.

Consumers’ satisfaction with service provided at the nearby health care facility was found to have a significant, positive association. This is comparable with previous finding [3, 12]. Obviously, satisfaction level of the client reflects the existing gap in the CBHI program implementation and client’s expectation, implying that clients with lower satisfaction were less likely to be enrolled in the CBHI scheme.

Age, gender, marital status, religion, area of residence and household income were shown to have significant association with enrollment in CBHI in previous studies [3, 29–31]. However, in this study they were not significantly associated with enrollment in CBHI. This could be due to socio- economic and cultural background difference of the study populations.

In this study, household wealth index is not significantly associated with direct enrollment in CBHI. Possible explanation for this may include; response bias due to respondent’s expectation of aid from both government and NGOs as Boricha district is known by dry weather and exclusively natural rain dependent. Thus, large numbers of dwellers in the district are supported by various NGOs. This indicates respondent’s expectation might make them to tell either under or overestimate assets they do have; on other hands, almost all households in the study area had cultivated lands and owned common domestic animals. This tells that there might not as such economic difference between the households. Thus, these reasons might have affected the association of household wealth index with enrollment in CBHI.

Our study is not without limitation however. Possibility of social desirability biases is one, though we attempted to provide clear information about the aim of the study, for example, a response to monthly income. As one would expect, possibility of adverse selection bias by scheme members is another limitation of this study. Third, we converted some original level information in to categorical information to present the results in a more unified format, for example the variables “time taken to reach nearby facility” beside its advantage, this might have limited the information we received from respondents.

## Conclusions and recommendations

This study revealed that the current enrollment status of households in CBHI is low in the study area. Consequently, the study identified family size, educational status of households’ head, having information about CBHI, timing of premium collection, satisfaction in the services, and lack of trust on scheme management and presence of chronic illness in the family as the associated factors with the low enrollment of CBHI in the study area. Therefore, more awareness creation, revising timing of premium collection in consideration with local context, improving scheme management system in increasing members’ trust should be emphasized to improve CBHI enrollment.

## Declaration

### Ethical approval

Ethical clearance and supportive letter were obtained from Hawassa University College of Medicine and Health Sciences Institutional Review Board (IRB). Also permission letter were taken from Hawassa University School of Public Health, Sidama Zone Health Department and Boricha District Health Office. To maintain confidentiality individual identifiers was excluded. Verbal informed consent was taken from each respondent after approval of IRB. Since the majority of the study population was from rural informal sectors, we anticipated literacy status of the study participants by verbal informed consent. This is also permissible by the IRB of Hawassa University College of Medicine and Health Sciences. However, participants were given full right to participate or not to participate fully or partially.

## Supporting information

S1 Data (SAV)

## List of abbreviations

AOR: Adjusted Odds Ratios
CBHIS: Community Based Health Insurance Scheme
CBHI: Community Based Health Insurance
CI: Confidence Interval
COR: Crude Odds Ratios
FMOH: Federal Ministry of Health
FGDs: Focused Group Discussions
HHH: Households Head
IRB: Institutional Review Board
MPHEP: Masters-Of Public Health in Epidemiology
NGO: Non-Governmental Organization
OOPs: Out-Of-Pockets
ORs: Odds Ratios
PCA: Principal Component Analysis
PPS: Proportional to the Size
PCU: Primary Care Unit
SNNPR: South Nation Nationalities People Region
SPSS: Statistical Package for Social Science
WHO: World Health Organization
